# Longitudinal alterations of pulmonary $\dot{\text{V}}$O_2_ on-kinetics during moderate-intensity exercise in competitive youth cyclists are related to alterations in the balance between microvascular O_2_ distribution and muscular O_2_ utilization

**DOI:** 10.3389/fspor.2022.982548

**Published:** 2022-11-16

**Authors:** Matthias Hovorka, Bernhard Prinz, Dieter Simon, Manfred Zöger, Clemens Rumpl, Alfred Nimmerichter

**Affiliations:** ^1^Training and Sports Sciences, University of Applied Sciences Wiener Neustadt, Wiener Neustadt, Austria; ^2^Centre for Sport Science and University Sports, University of Vienna, Vienna, Austria; ^3^Doctoral School of Pharmaceutical, Nutritional and Sport Sciences, University of Vienna, Vienna, Austria

**Keywords:** near-infrared spectroscopy, pulmonary kinetics, youth cyclists, longitudinal, oxidative phosphorylation, microvascular blood flow, oxygen uptake, muscular oxygen utilization

## Abstract

**Purpose:**

The main purpose of the current study was to investigate the dynamic adjustment of pulmonary oxygen uptake ($\dot{\text{V}}$O_2_) in response to moderate-intensity cycling on three occasions within 15 months in competitive youth cyclists. Furthermore, the muscle Δdeoxy[heme] on-kinetics and the Δdeoxy[heme]-to-$\dot{\text{V}}$O_2_ ratio were modeled to examine possible mechanistic basis regulating pulmonary $\dot{\text{V}}$O_2_ on-kinetics.

**Methods:**

Eleven cyclists (initial age, 14.3 ± 1.6 y; peak $\dot{\text{V}}$O_2_, 62.2 ± 4.5 mL.min^−1^.kg^−1^) with a training history of 2–5 years and a training volume of ~10 h per week participated in this investigation. $\dot{\text{V}}$O_2_ and Δdeoxy[heme] responses during workrate-transitions to moderate-intensity cycling were measured with breath-by-breath spirometry and near-infrared spectroscopy, respectively, and subsequently modeled with mono-exponential models to derive parameter estimates. Additionally, a normalized Δdeoxy[heme]-to-$\dot{\text{V}}$O_2_ ratio was calculated for each participant. One-way repeated-measures ANOVA was used to assess effects of time on the dependent variables of the responses.

**Results:**

The $\dot{\text{V}}$O_2_ time constant remained unchanged between the first (~24 s) and second visit (~22 s, *P* > 0.05), whereas it was significantly improved through the third visit (~13 s, *P* = 0.006–0.013). No significant effects of time were revealed for the parameter estimates of the Δdeoxy[heme] response (*P* > 0.05). A significant Δdeoxy[heme]-to-$\dot{\text{V}}$O_2_ ratio “overshoot” was evident on the first (1.09 ± 0.10, *P* = 0.006) and second (1.05 ± 0.09, *P* = 0.047), though not the third (0.97 ± 0.10, *P* > 0.05), occasion. These “overshoots” showed strong positive relationships with the $\dot{\text{V}}$O_2_ time constant during the first (*r* = 0.66, *P* = 0.028) and second visit (*r* = 0.76, *P* = 0.007). Further, strong positive relationships have been observed between the individual changes of the fundamental phase τ_p_ and the Δdeoxy[heme]-to-$\dot{\text{V}}$O_2_ ratio “overshoot” from occasion one to two (*r* = 0.70, *P* = 0.017), and two to three (*r* = 0.74, *P* = 0.009).

**Conclusion:**

This suggests that improvements in muscle oxygen provision and utilization capacity both occurred, and each may have contributed to enhancing the dynamic adjustment of the oxidative “machinery” in competitive youth cyclists. Furthermore, it indicates a strong link between an oxygen maldistribution within the tissue of interrogation and the $\dot{\text{V}}$O_2_ time constant.

## Introduction

The dynamic response of pulmonary oxygen uptake ($\dot{\text{V}}$O_2_) following a square-wave transition from rest to moderate-intensity [i.e., below the gas exchange threshold (GET)] constant-workrate exercise is characterized by three phases (pulmonary $\dot{\text{V}}$O_2_ on-kinetics). The first increase in pulmonary $\dot{\text{V}}$O_2_ during phase I (i.e., cardiodynamic phase) is largely dictated by a fast increase in cardiac output; and hence, pulmonary blood flow during the first 15–20 s of the transition. The subsequent exponential increase during phase II (i.e., fundamental phase) drives the pulmonary $\dot{\text{V}}$O_2_ toward its projected steady-state (phase III) (1, 2). The fundamental phase is described by the time constant (τ_p_), which (i) reflects the time to achieve 63% of the projected phase II response (3) and (ii) coincides within ~10% with a surrogate of muscular $\dot{\text{V}}$O_2_ (i.e., kinetics of muscle phosphocreatine breakdown) in children (4). Therefore, the fundamental phase τ_p_ can be used as a substitute of muscular $\dot{\text{V}}$O_2_ on-kinetics and provide useful information regarding the dynamic adjustment of the metabolic processes located in the working myocytes (3).

Pulmonary $\dot{\text{V}}$O_2_ on-kinetics during moderate-intensity exercise have been extensively studied in healthy and diseased adults, whereas data in (endurance trained) children and adolescents are limited (3, 5). Previous studies revealed no significant differences of the fundamental phase τ_p_ between prepubertal children and young adults (6–8), whereas more recent investigations showed smaller τ_p_ values (i.e., faster on-kinetics) in prepubertal children vs. young adults (9–12). Cross-sectional comparisons between endurance-trained and untrained youth reported either faster (13, 14) or similar (15) fundamental phase τ_p_ in the endurance-trained vs. untrained participants. However, to the best of the authors knowledge, no study has yet investigated longitudinal alterations of pulmonary $\dot{\text{V}}$O_2_ on-kinetics during moderate-intensity exercise in endurance trained youth. Furthermore, there has been considerable debate on the regulatory factors of the dynamic $\dot{\text{V}}$O_2_ response following a transition to moderate-intensity exercise between those favoring metabolic limitations and those supporting oxygen (O_2_) delivery limitations (16, 17). Technologies like portable near-infrared spectroscopy (NIRS) devices applied together with established methods (e.g., breath-by-breath spirometry) have been previously used to investigate muscle O_2_ delivery/utilization relationships [e.g., Δdeoxy[heme]-to-$\dot{\text{V}}$O_2_ ratio] in children /adolescents and adults (11, 13, 14, 18–20); and thus, have the potential to further strengthen the understanding of the mechanistic bases regulating (changes of) $\dot{\text{V}}$O_2_ on-kinetics (16, 21). For example, Marwood et al. (13) showed faster pulmonary $\dot{\text{V}}$O_2_ and capillary blood flow on-kinetics in trained vs. untrained adolescents, whereas no significant differences in Δdeoxy[heme] on-kinetics have been observed. The authors concluded that proportional enhancements in O_2_ delivery and utilization capacity determined the faster pulmonary $\dot{\text{V}}$O_2_ on-kinetics reported in the trained group (13). Further, Murias et al. (19, 20) revealed that training induced improvements of pulmonary $\dot{\text{V}}$O_2_ on-kinetics in adults are associated with a reduction of the Δdeoxy[heme]-to-$\dot{\text{V}}$O_2_ ratio; and thus, an improved balance between microvascular O_2_ distribution and local muscular O_2_ utilization.

The main purpose of the current study was to investigate changes of pulmonary $\dot{\text{V}}$O_2_ on-kinetics in response to moderate-intensity exercise in competitive youth cyclists over a period of 15 months, and to model Δdeoxy[heme] on-kinetics and the Δdeoxy[heme]-to-$\dot{\text{V}}$O_2_ ratio to examine possible mechanisms regulating (changes of) the adjustment of oxidative phosphorylation. We hypothesized a speeding over time of the pulmonary $\dot{\text{V}}$O_2_ on-kinetic response concomitant with no changes of the Δdeoxy[heme] on-kinetics. Additionally, we expected a reduction of the Δdeoxy[heme]-to-$\dot{\text{V}}$O_2_ “overshoot” with time and a positive relationship between (changes of) the Δdeoxy[heme]-to-$\dot{\text{V}}$O_2_ “overshoot” and fundamental phase τ_p_ for all occasions.

## Materials and methods

### Participants

Eight male and three female youth cyclists with a training history of 2–5 years participated in the current investigation. All cyclists performed a regular endurance-training volume of ~10 h per week throughout the study duration, were members of the junior national team, attended a local sports high school, and regularly competed at national and international level competitions in road cycling, mountain bike XC, and track cycling. The cyclists were part of the same training group, and the whole training process was supervised by one experienced coach who followed a polarized training intensity distribution approach throughout the study duration. Prior to the study, the participants and their legal guardians were informed of the experimental procedures and gave written informed consent to participate. All documents and procedures were submitted to, and approved by, the institutional review board and the study was conducted in accordance with the Declaration of Helsinki.

### Experimental design

Participants visited the laboratory twice within 2 weeks on three occasions within 15 months (Occasion 1: 1st month, Occasion 2: 8th month, Occasion 3: 15th month). Body mass and stature were measured with an electronic scale and stadiometer (Seca 813 and 213, Seca, Hamburg, Germany) and adipose tissue thickness (ATT) at the musculus vastus lateralis was determined using a skinfold caliper (Harpenden, Baty International, Burgess Hill, United Kingdom) before a graded ramp exercise test (GXT) was conducted during the first visit. On a subsequent visit, participants performed one square-wave transition from a baseline workrate to a workrate corresponding to the moderate-intensity domain. All tests were conducted on the participants own road bikes mounted to a Cyclus2 Ergometer (RBM Electronics, Leipzig, Germany). Participants were instructed to visit the laboratory in a fully rested state and to refrain from alcohol 24 h and caffeine 3 h prior to testing.

### Graded ramp exercise test

The GXT was conducted to determine peak workrate (W_peak_), peak oxygen consumption ($\dot{\text{V}}$O_2peak_), peak heart rate (HR_peak_), and the GET and the respiratory compensation point (RCP). After a 3 min baseline at 40 W, the workrate increased at a rate of 20 W.min^−1^ until the limit of tolerance. Participants were asked to maintain a cadence between 90 and 100 rpm during the GXT. They breathed through a low-resistance impeller turbine mounted on a face mask to continuously measure gas exchange and pulmonary ventilation with a portable open circuit spirometry (MetaMax 3B, Cortex Biophysik, Leipzig, Germany). The gas analysers were calibrated with gases of known concentrations [15.99 Vol% oxygen (O_2_), 4.99 Vol% carbon dioxide (CO_2_), Cortex Biophysik, Leipzig, Germany] and air flow and volume were calibrated with a 3-L syringe (Type M 9474-C, Cortex Biophysik, Leipzig, Germany). $\dot{\text{V}}$O_2peak_, HR_peak_ and peak respiratory exchange ratio (RER_peak_) were defined as the highest continuous 30 s average throughout the test. The V-slope method was used to determine the GET (22) which was subsequently visually verified by inspection of an increase of the ventilatory equivalent of O_2_, without a concomitant change of the ventilatory equivalent of CO_2_. RCP was determined as the first systematic decrease in end-tidal partial pressure of CO_2_ with a concomitant increase of the ventilatory equivalent of CO_2_. It was subsequently visually verified by inspection of the second disproportional increase in minute ventilation (23).

### Square-wave transition

The square-wave transition was conducted to determine pulmonary $\dot{\text{V}}$O_2_ and local muscular deoxygenation on-kinetics. The required workrate for the square-wave transition was determined after the completion of the GXT as 90% GET. A 3 min baseline at 40 W was followed by a step increase in workrate to moderate-intensity for 6 min and a cool-down of 3 min at 40 W. Participants were asked to maintain a cadence between 90 and 100 rpm during the test. Pulmonary ventilation and gas exchange were continuously measured breath-by-breath as described above. Local muscular deoxygenation of the right m. vastus lateralis was determined using a multi-distance continuous-wave NIRS device (PortaMon, Artinis, Elst, The Netherlands). The NIRS probe was covered in a transparent household plastic film and tightly taped on the cleaned and shaved belly of the muscle, midway between the lateral epicondyle of the femur and the greater trochanter. The probe was further fixed with an elastic bandage and covered with a black hose to minimize movement artifacts and the influence of extraneous light sources, respectively. The NIRS device consisted of three photodiodes emitting light at a wavelength of 762 to 850 nm and a photon detector detecting photons emerging form the interrogated tissue. Light source-detector distances of 30, 35, and 40 mm enabled a penetration depth of 15–20 mm. The device utilized the modified Beer-Lambert law to calculate relative changes of the local tissue deoxygenation status. The Δdeoxy[heme] signal was used for the “physiological calibration” described in the following paragraph.

Following the completion of the square-wave transition protocol, a(n) ischemia/hyperaemia calibration was conducted to normalize the Δdeoxy[heme] signal to its maximal “physiological” range. For this purpose, participants laid down on a massage table in a supine position. A blood pressure cuff (Ulrich medical, Ulm, Germany) attached to a cuff inflator (heidi™ mein Tourniquet, Ulrich medical, Ulm, Germany) was placed proximally of the NIRS probe and inflated to a pressure of ~300 mmHg for 5 min followed by an instantaneous release of the pressure. The Δdeoxy[heme] plateau during the ischemic phase and the Δdeoxy[heme] minimum during the hyperaemic phase of the calibration represents 100 and 0% deoxygenation in the tissue interrogated by the NIRS device. This “physiological calibration” allows the obtainment of “semiquantitative” tissue deoxygenation indices and thus the comparison between participants with differing [heme] and/or adipose tissue thickness (21). As suggested previously, this normalized Δdeoxy[heme] signal was used for further analysis (21).

### Data analysis

#### Pulmonary $\dot{\text{V}}$O_2_ on-kinetic data modeling

The pulmonary breath-by-breath $\dot{\text{V}}$O_2_ data were filtered by removing aberrant breaths that lay outside more than four standard deviations (SD) of the local mean of five data points. The filtered data then were linearly interpolated to receive second-by-second data. These 1-s interpolated data were time-aligned that time zero represents the onset of exercise for each individual. Data of the first 15 s of the square-wave transition were excluded from the analysis to account for the cardiodynamic phase (24, 25), and a mono-exponential model was applied to model the fundamental phase of the pulmonary $\dot{\text{V}}$O_2_ on-kinetics (Equation. 1).


(1) $$\begin{array}{l} \dot{V}O_{2}\left(t\right)=BL+A_{p}\;.\;\left(1-\;e^{-\frac{\left(t-TD_{p}\right)}{\tau_{p}}}\right) \end{array}$$

where $\dot{\text{V}}$O_2_ (*t*) represents the pulmonary $\dot{\text{V}}$O_2_ at a given time *t*, BL is defined as the mean pulmonary $\dot{\text{V}}$O_2_ between −60 and −10 s of baseline cycling, A_p_ is considered as the steady-state increase of pulmonary $\dot{\text{V}}$O_2_ above BL, TD_p_ is the time delay relative to the onset of exercise and τ_p_ represents the pulmonary $\dot{\text{V}}$O_2_ time constant. The data were modeled from 15 s to the end of the exercise. The parameter estimates were subsequently estimated by least-squares non-linear regression analysis (GraphPad Prism 9.1.2, GraphPad Software Inc., San Diego, USA).

#### Δdeoxy[heme] on-kinetic data modeling

The normalized Δdeoxy[heme] data were averaged to 1-s bins and left-shifted that time zero represents the onset of exercise and subsequently modeled with a mono-exponential model (Equation 2). The start of the exponential increase was identified as the time at which the Δdeoxy[heme] signal started to systematically increase by one SD above baseline (18). Data were fitted up to 140 s, or, where a Δdeoxy[heme] overshoot relative to end-exercise was identified visually, to the peak value of this overshoot (18).


(2) $$\begin{array}{l} \Delta \text{deoxy}\left[\text{heme}\right]\left(t\right)=A_{m}\;.\;\left(1-\;e^{-\frac{\left(t-TD_{m}\right)}{\tau_{m}}}\right) \end{array}$$

where Δdeoxy[heme] (*t*), A_m_, TD_m_, and τ_m_ represent the tissue deoxygenation status at any time *t*, the asymptotic amplitude, the time delay and the time constant of the Δdeoxy[heme] response, respectively. The MRT_m_ was calculated as the sum of TD_m_ and τ_m_.

#### Δdeoxy[heme]-to-$\dot{\text{V}}$O_2_ ratio modeling

In addition to the on-kinetic responses, a normalized Δdeoxy[heme]-to-$\dot{\text{V}}$O_2_ ratio was derived from the actual data profiles of pulmonary $\dot{\text{V}}$O_2_ and Δdeoxy[heme] for each individual. A ratio of 1.00 represents a steady-state value between O_2_ delivery and utilization, whereas an “overshoot” beyond values of 1.00 indicates a slower adjustment of microvascular O_2_ delivery in proportion to the O_2_ demand; and hence, is thought to represent a temporary maldistribution of O_2_ within the working muscles (17, 19, 26, 27). Briefly, the second-by-second pulmonary $\dot{\text{V}}$O_2_ and Δdeoxy[heme] data were normalized that 0 % corresponds to the baseline values and 100% reflects the steady-state response of pulmonary $\dot{\text{V}}$O_2_ and Δdeoxy[heme]. To account for the cardiodynamic phase, the normalized pulmonary $\dot{\text{V}}$O_2_ data were time-aligned that time zero represents the onset of the fundamental phase of the pulmonary $\dot{\text{V}}$O_2_ response. Subsequently, the data were averaged to 5-s bins and a mean normalized Δdeoxy[heme]-to-$\dot{\text{V}}$O_2_ ratio was calculated for each individual from 15 to 120 s (26). The start and end point of 15 and 120 s coincide with the start of the ratio “overshoot” and the point at which all participants Δdeoxy[heme] and pulmonary $\dot{\text{V}}$O_2_ responses reached their amplitude, respectively.

### Statistical analyses

Descriptive data are presented as mean ± SD. Shapiro-Wilk and Mauchly tests were used to examine assumptions of normality and sphericity, respectively. One-way repeated-measures ANOVA were used to determine possible effects of time on the dependent variables of the pulmonary $\dot{\text{V}}$O_2_ and Δdeoxy[heme] on-kinetic responses and the results of the GXT. Bonferroni correction was used for pairwise comparisons where appropriate. *T*-tests were applied to assess a significant “overshoot” (i.e., >1.00) of the normalized Δdeoxy[heme]-to-$\dot{\text{V}}$O_2_ ratio (one sample *t*-test). Pearson's product moment correlations were used to determine the relationship between the normalized Δdeoxy[heme]-to-$\dot{\text{V}}$O_2_ ratio and the fundamental phase τ_p_. All statistical and graphical analyses were performed using IBM SPSS Statistics 26 (SPSS Inc., Chicago, IL, USA) and GraphPad Prism 9.1.2 (GraphPad Software Inc., San Diego, CA, USA), respectively. The level of statistical significance was set at *P* ≤ 0.05.

## Results

Participants characteristics and results of the GXT are presented in Table 1. The one-way repeated-measures ANOVA revealed significant effects of time on stature [*F*_(1.14,11.43)_ = 10.579, *P* = 0.006], body mass [*F*_(1.14,11.41)_ = 11.284, *P* = 0.005], W_peak_ [*F*_(1.22,12.24)_ = 9.119, *P* = 0.008], the workrate corresponding to 90% GET [*F*_(1.18,11.81)_ = 6.996, *P* = 0.018] and RCP [*F*_(1.16,11.56)_ = 13.685, *P* = 0.003], absolute $\dot{\text{V}}$O_2_ at GET [*F*_(1.29,12.91)_ = 7.122, *P* = 0.015] and RCP [*F*_(1.22,12.15)_ = 8.189, *P* = 0.011], relative $\dot{\text{V}}$O_2_ at RCP [*F*_(2,20)_ = 8.398, *P* = 0.002], and RER_peak_ [*F*_(2,20)_ = 4.218, *P* = 0.030], whereas no significant effect of time was reported on the remaining parameters (*P* = 0.116–0.724). The pairwise comparisons revealed increases in stature and body mass from the first to the second (*P* = 0.002 and 0.005 for stature and body mass, respectively) and third occasion (*P* = 0.011 and 0.008 for stature and body mass, respectively). Further, W_peak_ increased from occasion one to two (*P* = 0.004) and three (*P* = 0.022), and absolute $\dot{\text{V}}$O_2_ at GET and RCP increased from occasion two compared to three (*P* = 0.035 and *P* = 0.029, respectively). Workrate at RCP increased from occasion one to two (*P* = 0.006) and three (*P* = 0.006), and from occasion two to three (*P* = 0.038), while relative $\dot{\text{V}}$O_2_ at RCP increased from occasions one/two compared to three (*P* = 0.045 and *P* = 0.027, respectively). Furthermore, pairwise comparisons revealed a significant difference between RER_peak_ at occasion one and two (*P* = 0.005). However, no significant differences in workrates corresponding to 90% GET have been found between any test occasions (*P* = 0.054–0.316).

**Table 1:** Participants characteristics and results of the graded ramp exercise test as mean ± SD (*n* = 11).

	**Occasion 1**	**Occasion 2**	**Occasion 3**
Age (y)	14.3 ± 1.6	15.0 ± 1.6^*^	15.6 ± 1.6^*^, ^**^
Stature (cm)	163.1 ± 12.9	165.3 ± 12.9^*^	168.5 ± 12.1^*^
Body mass (kg)	52.7 ± 12.1	54.3 ± 12.2^*^	57.6 ± 11.3^*^
ATT m. vastus lateralis (mm)	5.2 ± 1.5	5.3 ± 1.3	6.4 ± 1.4
Workrate 90% GET (W)	125 ± 25	128 ± 25	137 ± 24
$\dot{\text{V}}$O_2_ at GET (mL.min^−1^)	1,780 ± 394	1,800 ± 407	2,021 ± 380^**^
$\dot{\text{V}}$O_2_ at GET (%$\dot{\text{V}}$O_2peak_)	54.9 ± 4.8	52.8 ± 3.3	57.3 ± 6.9
Workrate RCP (W)	215 ± 45	229 ± 44^*^	258 ± 55^*^, ^**^
$\dot{\text{V}}$O_2_ at RCP (mL.min^−1^)	2,616 ± 586	2,650 ± 561	3,036 ± 688^**^
$\dot{\text{V}}$O_2_ at RCP (%$\dot{\text{V}}$O_2peak_)	80.4 ± 4.3	77.9 ± 6.7	85.1 ± 6.9^*^, ^**^
HR_peak_ (beats.min^−1^)	197 ± 5	196 ± 5	195 ± 7
RER_peak_	1.21 ± 0.03	1.17 ± 0.03^*^	1.19 ± 0.06
W_peak_ (W)	290 ± 54	308 ± 59^*^	324 ± 64^*^
$\dot{\text{V}}$O_2peak_ (mL.min^−1^.kg^−1^)	62.2 ± 4.5	63.1 ± 6.1	62.0 ± 6.0
$\dot{\text{V}}$O_2peak_ (mL.min^−1^)	3,259 ± 728	3,409 ± 746	3,551 ± 669

SD, standard deviation; ATT, adipose tissue thickness; GET, gas exchange threshold; HR_peak_, peak heart rate; RCP, respiratory compensation point; RER_peak_, peak respiratory exchange ratio; W_peak_, peak workrate; $\dot{\text{V}}$O_2peak_, peak oxygen consumption.

*Post-hoc* tests:

* Significantly different from test occasion 1 (P < 0.05),

** significantly different from test occasion 2 (P < 0.05).

### Pulmonary $\dot{\text{V}}$O_2_ and muscular Δdeoxy[heme] on-kinetics

**Figure 1:**
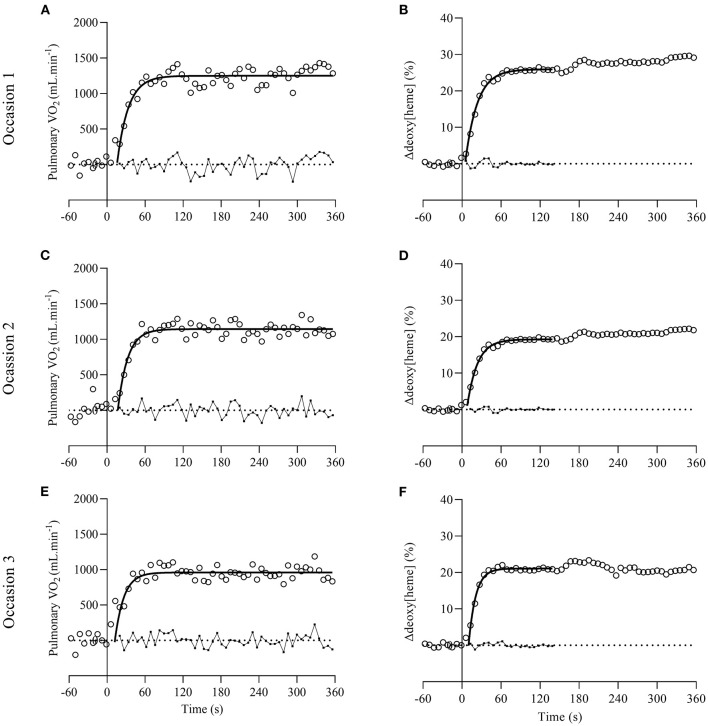
Representative plots of pulmonary $\dot{\text{V}}$O_2_ [open circles on **(A,C,E)**] and muscular Δdeoxy[heme] on-kinetics [open circles on **(B,D,F)**] from one participant on occasion one, two, and three. Dotted lines represent baselines where pulmonary $\dot{\text{V}}$O_2_ and Δdeoxy[heme] is zero. Solid lines and small full circles with connective lines represent the exponential curve fits and residuals, respectively.

Figure 1 shows representative plots of the pulmonary $\dot{\text{V}}$O_2_ [panel **(A,C,E)**] and muscular Δdeoxy[heme] on-kinetics [panel **(B,D,F)**] from one participant. A significant effect of time was revealed for the fundamental phase τ_p_ [*F*_(2,20)_ = 9.776, *P* = 0.001] of the pulmonary $\dot{\text{V}}$O_2_ on-kinetic response (Table 2). *Post-hoc* tests showed that the fundamental phase τ_p_ was significantly smaller (i.e., faster) on occasion three (12.9 ± 4.8 s) compared with occasion one (24.2 ± 6.6 s, *P* = 0.006) and occasion two (21.7 ± 6.0 s, *P* = 0.013). The one-way ANOVA revealed no significant effect of time for all parameter estimates describing the muscular Δdeoxy[heme] on-kinetic response to a moderate-intensity square-wave transition (*P* = 0.111–0.671; Table 2).

**Table 2:** Pulmonary $\dot{\text{V}}$_2_ and Δdeoxy[heme] on-kinetic parameter in response to a square-wave transition to the moderate-intensity domain as mean ± SD (*n* = 11).

	**Occasion 1**	**Occasion 2**	**Occasion 3**
**Pulmonary** $\dot{\text{V}}$O_**2**_ **on-kinetic**			
A_p_ (mL.min^−1^)	687 ± 277	664 ± 239	743 ± 238
Gain (mL.min^−1^.W^−1^)	10.6 ± 1.0	9.9 ± 0.8	9.7 ± 0.9
TD_p_ (s)	10.5 ± 2.8	12.4 ± 3.6	12.0 ± 4.4
τ_p_ (s)	24.2 ± 6.6	21.7 ± 6.0	12.9 ± 4.8^*^, ^**^
CI_95_ τ_p_ (s)	4.6 ± 2.2	5.3 ± 2.3	4.1 ± 1.4
**Muscular** **Δdeoxy[heme] on-kinetic**			
A_m_ (%)	13.7 ± 9.3	10.9 ± 3.5	11.3 ± 5.3
TD_m_ (s)	7.3 ± 1.9	10.4 ± 4.6	9.7 ± 1.7
τ_m_ (s)	11.2 ± 3.9	11.6 ± 3.4	11.2 ± 4.9
CI_95_ τ_m_ (s)	3.7 ± 2.8	2.3 ± 0.7	2.0 ± 1.1
MRT_m_ (s)	18.5 ± 3.6	22.1 ± 4.5	20.9 ± 5.7
**Normalized Δdeoxy[heme]/**$\dot{\text{V}}$**O**_**2**_ **ratio**	1.09 ± 0.10^$^	1.05 ± 0.09^$^	0.97 ± 0.10^*^

SD, standard deviation; A, amplitude of the response; TD, time delay; τ, time constant; CI_95_, 95% confidence interval; MRT, mean response time.

*Post-hoc* tests:

* Significantly different from test occasion 1 (P < 0.05),

** significantly different from test occasion 2 (P < 0.05).

$ Significantly higher than 1.00 (P < 0.05).

### Δdeoxy[heme]-to-$\dot{\text{V}}$O_2_ ratio

**Figure 2:**
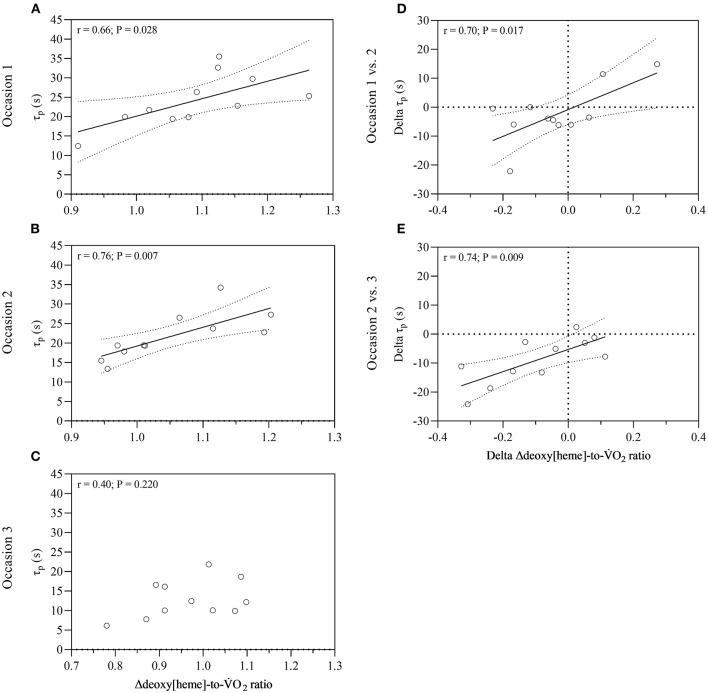
Relationship between the normalized Δdeoxy[heme]-to-$\dot{\text{V}}$O_2_ ratio and fundamental phase τ_p_ for all three occasions **(A–C)**, and between changes of the normalized Δdeoxy[heme]-to-$\dot{\text{V}}$O_2_ ratio and fundamental phase τ_p_ over time **(D,E)**. Dotted lines represent 95% confidence bands. $\dot{\text{V}}$O_2_, pulmonary oxygen uptake; τ_p_, time constant of pulmonary oxygen uptake; r, coefficient of correlation.

A significant effect of time on the normalized Δdeoxy[heme]-to-$\dot{\text{V}}$O_2_ ratio was revealed [*F*_(2,20)_ = 4.717, *P* = 0.021]. *Post-hoc* tests showed that the ratio was lower on occasion three compared to one (*P* = 0.021). The normalized Δdeoxy[heme]-to-$\dot{\text{V}}$O_2_ ratio was significantly higher than 1.00 on test occasion one (1.09 ± 0.10, *P* = 0.006) and two (1.05 ± 0.09, *P* = 0.047), whereas it was not significantly higher on occasion three (0.97 ± 0.10, *P* = 0.151; Table 2). The Δdeoxy[heme]-to-$\dot{\text{V}}$O_2_ ratio showed a strong positive relationship with the fundamental phase τ_p_ on test occasion one (*r* = 0.66, *P* = 0.028) and two (*r* = 0.76, *P* = 0.007), though this relationship was not significant on occasion three (*r* = 0.40, *P* = 0.220; Figures 2A–C). Further, a strong positive relationship was observed between the change of the fundamental phase τ_p_ and the Δdeoxy[heme]-to-$\dot{\text{V}}$O_2_ ratio from occasion one to two (*r* = 0.70, *P* = 0.017), and two to three (*r* = 0.74, *P* = 0.009; Figures 2D,E).

## Discussion

The present study examined longitudinal changes in pulmonary $\dot{\text{V}}$O_2_ and Δdeoxy[heme] on-kinetics, and the Δdeoxy[heme]-to-$\dot{\text{V}}$O_2_ ratio in response to moderate-intensity exercise in trained youth cyclists over a period of 15 months. The main findings were: (i) Partially in line with our hypothesis, the fundamental phase τ_p_ showed no significant change from the first to the second visit, whereas τ_p_ decreased significantly from the first/second to the third visit. (ii) In line with our hypothesis, no significant changes of the Δdeoxy[heme] on-kinetic parameter estimates were observed during the current investigation. (iii) A transient Δdeoxy[heme]-to-$\dot{\text{V}}$O_2_ overshoot relative to the steady-state value of ~1.00 was present on test occasion one and two, whereas this overshoot was abolished on occasion three. (iv) A strong positive relationship between the Δdeoxy[heme]-to-$\dot{\text{V}}$O_2_ ratio overshoot and the fundamental phase τ_p_ was revealed during the first and second visit, though this relationship was attenuated during the third visit. (v) A strong positive correlation was observed between the change of the fundamental phase τ_p_ and the Δdeoxy[heme]-to-$\dot{\text{V}}$O_2_ ratio from occasion one to two, and two to three.

### Longitudinal changes of the on-kinetic responses

The fundamental phase τ_p_ reported on test occasions one and two (~24 and ~22 s, respectively) are in line with previous investigations in endurance-trained adolescents of similar age (~22–26 s) (13, 14). However, the τ_p_ reported on test occasion three (~13 s) is well below these values (i.e., faster) and coincides with $\dot{\text{V}}$O_2_ on-kinetics found in well- to highly-trained adult cyclists, rowers or runners (28–33), and a Belgian Junior cycling champion (3). Due to the lack of a control group in the present investigation, it is difficult to interpret whether the observed speeding of the fundamental phase τ_p_ may be attributed to the endurance training performed by the youth cyclists. Previous studies have shown that the fundamental phase τ_p_ is either faster (9–12) or similar (6–8) in untrained prepubertal children compared with untrained young adults. Thus, it seems likely to suggest that the herein reported speeding of the $\dot{\text{V}}$O_2_ on-kinetic response may be largely ascribed to the endurance training performed by the youth cyclists. The notion of a trainable on-kinetic response in youth is further supported by investigations revealing faster pulmonary $\dot{\text{V}}$O_2_ on-kinetics in trained vs. untrained youth (13, 14).

The time course for the dynamic adjustment of the Δdeoxy[heme] signal (i.e., τ_m_ and MRT_m_) remained constant throughout the study. This in in line with previous cross-sectional studies reporting no significant differences in Δdeoxy[heme] on-kinetics between trained and untrained adolescents (13, 14). In concert with the speeding of the fundamental phase τ_p_, this indicates a proportional enhancement of microvascular O_2_ provision and O_2_ utilization capacities (13, 14) between test occasion one/two and three herein. This is supported by studies showing faster heart rate on-kinetics, indicative of an enhanced bulk blood flow, in trained vs. untrained prepubertal children (13), and an increase in muscle oxidative capacity in response to endurance training in youth (34, 35). However, it is noteworthy to mention that an elevated bulk blood flow does not ultimately mean that there was a faster local O_2_ distribution. Overall, it may be suggested that improvements in local muscular O_2_ distribution and O_2_ utilization capacities both occurred, and each may have contributed to improving the pulmonary $\dot{\text{V}}$O_2_ on-kinetic response observed herein. Again, due to the lack of a control group it is difficult to interpret whether these adaptations may be attributed to exercise training. However, since previous studies reported a higher percentage of type I muscle fibers (36) and faster capillary blood flow kinetics (11) in male children/adolescents compared to adults, and an elevated oxidative enzyme content (37) in male and female adolescents compared to adults, it seems appropriate to associate the above-mentioned adaptations with the exercise training performed by the youth cyclists in the current study.

### Possible mechanistic basis

The Δdeoxy[heme] signal showed a TD_m_ of ~7–10 s during the early phase of the transient which was not affected by time in the present investigation. This is in line with previous investigations showing similar TD_m_ values (~7–9 s) in adolescents which were not affected by training status and/or age (11, 13, 14). The steady Δdeoxy[heme] signal during the early phase of the exercise transition suggests a precise matching of local O_2_ distribution to utilization in the area of interrogation (16). This notion is in line with studies showing a similar pattern of O_2_ distribution/utilization indices (i.e., intracellular PO_2_, arterio-venous O_2_ difference) in animal myofiber preparations (38–40) and human limbs (41). Since muscle $\dot{\text{V}}$O_2_ increases immediately after the onset of exercise (41, 42), a concomitant instant increase in local O_2_ distribution is mandatory to preserve this early “steady-state.” Such a rapid increase in capillary blood flow; and thus, microvascular O_2_ delivery, has been previously shown early during the transient (43, 44). Together, this indicates intracellular mechanisms other than regional O_2_ maldistribution to constrain the adjustment of oxidative phosphorylation during the first ~10 s of the transient.

Following this early “homeostasis” between microvascular O_2_ provision and O_2_ demand within the working myofibers, Δdeoxy[heme] increased exponentially, and a Δdeoxy[heme]-to-$\dot{\text{V}}$O_2_ overshoot was evident on test occasion one and two, though not on occasion three. The Δdeoxy[heme]-to-$\dot{\text{V}}$O_2_ overshoot has been previously interpreted as a greater reliance on O_2_ extraction in proportion to the O_2_ demand within the muscle tissue (17, 19, 26, 27). Together, this indicates that on average a temporal mismatch between local O_2_ distribution and O_2_ demand following the first ~10 s after exercise onset is evident on occasion one and two, though not three. A mitigated or abrogated Δdeoxy[heme]-to-$\dot{\text{V}}$O_2_ overshoot indicates a reduced reliance on O_2_ extraction and thus, a more precise matching between microvascular O_2_ provision and utilization within the tissue of interrogation (17, 19, 26, 27). This may result in a less pronounced fall in microvascular PO_2_; and hence, an elevated driving force regulating the capillary-to-myocyte O_2_ flux resulting in a higher potential for oxidative phosphorylation during the transition (45, 46). This is supported by: (i) The strong positive relationships observed between the extent of the Δdeoxy[heme]-to-$\dot{\text{V}}$O_2_ overshoot and the fundamental phase τ_p_ on occasion one and two (Figures 2A–C), and by the fast τ_p_ observed on occasion three where the Δdeoxy[heme]-to-$\dot{\text{V}}$O_2_ overshoot and hence, an O_2_ maldistribution, was abrogated. (ii) The strong positive correlation between the change of the Δdeoxy[heme]-to-$\dot{\text{V}}$O_2_ ratio and the fundamental phase τ_p_ from occasion one to two, and two to three (Figures 2D,E).

### Limitations

One limitation resides in the NIRS measurement *per-se* (e.g., probe placement, small tissue of interrogation). To at least partially counteract these issues, we implemented a standardized operating procedure regarding probe placement to minimize the influence of spatial heterogeneities within the tissue of interest and followed the specific recommendations recently stated by Barstow (21). Limitations related to the modeling of the Δdeoxy[heme]-to-$\dot{\text{V}}$O_2_ ratio have been discussed extensively elsewhere (17, 21, 26). Briefly, modeling simulations revealed that the currently used method is rather conservative in estimating the “overshoot;” and hence, conclusions would have been unaffected by using another modeling approach (26). The use of only one exercise transition may be considered as a further limitation. Recent studies have shown that multiple transitions increase the confidence in the parameter estimates of the $\dot{\text{V}}$O_2_ and Δdeoxy[heme] on-kinetics (25, 47) and thus, decrease the smallest change detectable with confidence. However, the herein reported 95% confidence intervals for the fundamental phase τ_p_ (~4–5 s) and τ_m_ (~2–4 s) are within acceptable boundaries (13, 14), and the mean ~8–11 s decrease in τ_p_ between occasion one/two and three is at least similar to the smallest change detectable with confidence in youth by using one exercise transition (47). The lack of a control group may be considered another limitation. However, since previous investigations showed a slowing or no change of the pulmonary $\dot{\text{V}}$O_2_ on-kinetic response with aging, and a lower potential for oxidative metabolism (e.g., % type I fibers and/or oxidative enzyme content) in adults vs. youth [for review see: (5)] it seems appropriate to attribute the speeding of the pulmonary $\dot{\text{V}}$O_2_ on-kinetic response reported herein to the endurance-training performed by the youth cyclists.

## Conclusion

The data of the current investigation in competitive youth cyclists showed that the fundamental phase τ_p_ and hence, muscle $\dot{\text{V}}$O_2_ on-kinetics, was not affected by time from the first to the second, though from the first/second to the third visit. Concomitant with the unchanged Δdeoxy[heme] on-kinetics, this indicates a proportional improvement in muscle O_2_ distribution and O_2_ utilization capacity between the second and third visit, and both may have contributed to improve the pulmonary $\dot{\text{V}}$O_2_ on-kinetic response observed herein. Furthermore, the data presented herein indicate a strong link between an O_2_ maldistribution within the tissue of interrogation evident during exercise transitions on occasion one and two, and the fundamental phase τ_p_ in trained youth cyclists.

## Data availability statement

The raw data supporting the conclusions of this article will be made available by the authors, without undue reservation.

## Ethics statement

The studies involving human participants were reviewed and approved by Review Board, University of Applied Sciences Wiener Neustadt, Wiener Neustadt, Austria. Written informed consent to participate in this study was provided by the participants' legal guardian/next of kin.

## Author contributions

AN conceived and designed the research. BP, CR, MH, and MZ conducted the experiments. DS, MH, and MZ analyzed the data. DS and MH interpreted the results of the experiments. AN, BP, and MH drafted the manuscript. All authors were involved in the revision and approval of the final version of the manuscript.

## Funding

This research was supported by Gesellschaft für Forschungsförderung Niederösterreich m.b.H. (Grant No. SC18-014).

## Conflict of interest

The authors declare that the research was conducted in the absence of any commercial or financial relationships that could be construed as a potential conflict of interest.

## Publisher's note

All claims expressed in this article are solely those of the authors and do not necessarily represent those of their affiliated organizations, or those of the publisher, the editors and the reviewers. Any product that may be evaluated in this article, or claim that may be made by its manufacturer, is not guaranteed or endorsed by the publisher.
